# Transdiagnostic considerations are critical to understanding childhood neurodevelopmental disorders

**DOI:** 10.3389/fnhum.2024.1385873

**Published:** 2024-05-07

**Authors:** Betsy Hoza, Erin K. Shoulberg

**Affiliations:** Department of Psychological Science, University of Vermont, Burlington, VT, United States

**Keywords:** transdiagnostic, neurodevelopmental disorders, early childhood, assessment, screening, developmental delay, language, social functioning

## Abstract

Growing dissatisfaction with the current categorical diagnostic systems has led to a movement toward transdiagnostic dimensional approaches to assessment of childhood mental health disorders. We argue that a transdiagnostic approach is especially important and appropriate when screening for neurodevelopmental disorders during early childhood. In the early childhood years, symptoms often appear in the form of developmental delays that could portend a variety of different disorders. Early intervention at this point is critical, even though a final endpoint disorder is not yet apparent. Intervening early has the potential to grow the area of weakness, possibly correcting or at least ameliorating these delays. Early intervention requires a multidisciplinary approach integrating efforts across settings and providers that monitor the development of young children. We argue here that young children’s language ability is central to the development of social cognition, and a prerequisite for adequate social functioning. Social deficits are defining features of a subset of neurodevelopmental disorders such as autism spectrum disorder and social (pragmatic) communication disorder. Critically, impairment in social functioning is common in additional neurodevelopmental disorders such as attention-deficit/hyperactivity disorder (ADHD), learning disorders, and even motor disorders. For this reason, we argue that, at the earliest sign of a possible neurodevelopmental disorder, children should be screened for language deficits prior to initiating a focused assessment for a specific type of neurodevelopmental disorder such as ADHD. Any detected language deficits should be considered in the design and implementation of the assessment, as well as the ultimate intervention plan.

## 1 Introduction

A rising emphasis on dimensional conceptualizations of children’s mental health has emerged over the past 15 years (Pacheco et al., 2022). This evolution is a result of numerous developments, two of which are particularly relevant to our discussion of childhood neurodevelopmental disorders. First and foremost, there is a growing dissatisfaction with the categorical nature of our current diagnostic systems (Lahey, 2021; Astle et al., 2022). This dissatisfaction is fueled, at least in part, by the recognition of biological and psychosocial risk factors common across purportedly distinct disorders, the heterogeneity within supposedly unitary disorders, and the ebb and flow of symptoms that render rigid cut-points unsatisfactory for disorders that persist over much of the life span (Dalgleish et al., 2020).

Second, neurodevelopmental disorders emerged as a separate category for the first time in the Diagnostic and Statistical Manual of Mental Disorders, Fifth Edition (DSM-5; American Psychiatric Association, 2013), bringing together autism spectrum disorder (ASD), attention-deficit/hyperactivity disorder (ADHD), communication disorders, specific learning disorder, motor disorders, and intellectual developmental disorders. These disorders were united under the single umbrella category of neurodevelopmental disorders in the DSM-5 given that all are thought to be due to atypical brain development, and all are identifiable in early childhood (American Psychiatric Association, 2022). There are a number of reasons why this development was significant, from both theoretical and clinical perspectives. This unification emphasized commonalities among these disorders, some of which, previously, had been diagnostic exclusions for one another. For example, ASD and ADHD, no longer diagnostic exclusions, could now be diagnosed as comorbid disorders (Mikami et al., 2019). The umbrella category of neurodevelopmental disorders also allowed for a dimensional mapping of common domains of dysfunction across disorders, that, when considered together, explain potential impairments common to subsets of neurodevelopmental disorders (Gillberg, 2010; Astle et al., 2022). For example, delays or dysfunction in basic developmental domains (e.g., cognitive, language) may portend later impairments across a range of capacities such as attention, response inhibition, and communication, to name a few.

Importantly, in the earliest childhood years, initial concerns regarding neurodevelopmental disorders are usually raised in everyday clinical or educational settings such as pediatric or family medicine offices, or early childhood care and education settings (Lipkin et al., 2020). Hence, the most useful transdiagnostic domains for clinical care may be those that are accessible and intuitively relevant to professionals from a variety of disciplines and settings, as well as to parents, teachers, and other daily life caregivers, all of whom serve as monitors of development and initiators of services. Ideally, the domains should be tied to important developmental tasks of childhood, presented in a universally understood language, and observable at an early age. Therefore, we argue here that in the early childhood years (i.e., birth to age 8), the most useful transdiagnostic domains might reasonably coincide with the basic domains of child development. Broadly speaking, the primary tasks of early childhood involve developmental accomplishments related to cognitive, language, motor, social, behavioral, and emotional competence. Notably, this list of domains aligns considerably (though not exactly) with that proposed by Gillberg’s (2010) ESSENCE (Early Symptomatic Syndromes Eliciting Neurodevelopmental Clinical Examinations) approach. It also aligns considerably with the screening recommendations of the American Academy of Pediatrics (Lipkin et al., 2020).

Of note, a transdiagnostic dimensional approach provides a potential path forward in terms of developmental screening and early intervention whereby the earliest indicators of impairment (for example, delays in the basic developmental domains listed above) might be identified and targeted, prior to becoming full blown and impairing disorders (Astle et al., 2022; Sawrikar et al., 2022). Additionally, this transdiagnostic dimensional perspective acknowledges the likelihood that common underlying biological and psychosocial factors interact to become different phenotypes of disorder (Dalgleish et al., 2020). The beauty of this perspective is that it is inherently developmental. Specifically, it emphasizes expected change in presentation over time and allows for proactive intervention during periods of greater brain plasticity in early childhood to promote positive developmental trajectories (Astle et al., 2022). Importantly, it is also intentionally at odds with a “wait-and-see approach”, now considered outdated (Capone Singleton, 2018), and instead, encourages action whenever a concern is present (Gillberg, 2010).

## 2 Relevance to a special issue on social cognition and discourse processing?

Both social cognition and discourse processing are advanced transdiagnostic constructs that depend on functional capacity in several of the basic developmental domains listed above. At a minimum, the relevant domains are cognitive, language, and social. Social cognition is a very broad term that subsumes constructs related to how individuals perceive, process, interpret, and respond to social stimuli in their surroundings (Beaudoin and Beauchamp, 2020). Discourse skills, such as those needed to appropriately initiate, respond, and participate effectively in a conversation or other social interaction, are important to communicative competence (Westby, 2020) and, critically, related to social status with peers (van der Wilt et al., 2019).

It is possible, however, that both social cognition and discourse difficulties may be related to one or more impairments in more basic processes, such as working memory (WM). For example, Schuh et al. (2016) suggest the possibility “that WM deficits in ASD could lead to a cascade of effects whereby a) difficulties updating common ground knowledge b) lead to perspective-taking difficulties, which in turn c) impacts [sic] pragmatic language, including topic maintenance, reciprocal communication, and descriptive language” (p. 1349). Similarly, Du Bois et al. (2014) posit that early difficulties individuals with ASD experience with engaging caretakers and other interactional partners likely compromise linguistic development both in terms of understanding and producing language; this in turn, may hamper development of crucial discourse skills such as “[d]ialogic resonance [which] regularly occurs when one speaker draws on a prior utterance by a conversational partner as a resource for constructing a new utterance…” (p. 412). Relatedly, Tantucci and Wang (2023) report that children with ASD, relative to typically developing children, may have difficulty with the simultaneous partitioning of cognitive resources to both interactional engagement and creating new dialogic input in an ongoing interaction.

Hence, whereas both language skills and social cognition are key to successful social interactions during typical development, they are also hard to disentangle. In recent theoretical work, Rubio-Fernandez (2024) argues that language and social cognition “are connected in a positive feedback loop, whereby the development of one cognitive skill boosts the development of the other” (p. 18). Critically, we now know that difficulties with learning language in the early childhood years often portend difficulties in multiple life areas (e.g., educational, social, mental health) that persist across the life span (Reilly and McKean, 2023), making language difficulties a critical target for early intervention.

Importantly, social deficits are defining features of a subset of neurodevelopmental disorders such as ASD and social (pragmatic) communication disorder (American Psychiatric Association, 2022). However, even when not defining characteristics, social interaction difficulties are a common associated feature of other disorders such as ADHD (Hoza et al., 2005; Cervantes et al., 2013; Mikami et al., 2019), learning disorders (Nowicki, 2003; Milligan et al., 2016), and language disorders (Gertner et al., 1994; Janik Blaskova and Gibson, 2021; Wren et al., 2023). Given the pervasiveness of social difficulties across the neurodevelopmental disorders, and the centrality of language to social interactions, we argue for the need to screen for language delays or impairments whenever assessing for neurodevelopmental disorders.

## 3 Importance of language in assessing for childhood neurodevelopmental disorders

One topic not discussed often enough in the literature is the need to understand and consider a child’s level of language proficiency prior to utilizing a standardized child-focused assessment (Cormier et al., 2022). Standardized tests vary widely in the linguistic demands of their oral instructions (Graves et al., 2023). Even supposed nonverbal or spatial subtests rely to some extent on children’s receptive language proficiency to understand directions (Cormier et al., 2016). As argued by Cormier et al. (2022), failure to screen for language proficiency prior to undertaking a comprehensive assessment utilizing standardized tests has the potential to bias results and lead to incorrect estimations of cognitive ability. This bias is especially relevant for children whose first language is not English who may be less likely to fully understand the test instructions (Cormier et al., 2022), or for children from lower socioeconomic status backgrounds who may live in less language-enriched environments (Graves et al., 2023). Crucially, if language delay or impairment is misattributed as low cognitive ability due to the language-dependent nature of standardized tests, this misattribution can set the child on a course of low teacher expectations for attainment in school, which may, eventually, become a self-fulfilling prophesy (Gentrup et al., 2020). In addition, as noted by Benson and colleagues, despite limited empirical support, some schools still utilize an ability-achievement discrepancy model to qualify children for specific learning disorders diagnoses. Such models rely on a large magnitude difference between measured intelligence (i.e., IQ) and a subject area norm-referenced score to conclude that achievement is not commensurate with ability (Benson et al., 2020). In these situations, an underestimation of cognitive ability due to failure to screen for language proficiency can result in invalid placement decisions and inaccurate determinations regarding needed services (Benson et al., 2020; Graves et al., 2023). These practices can have a profound negative effect on a child’s school performance and achievement as they may continue, over time, to be denied needed services. Hence, our view is that it is critical to do a language screening on all children referred for a child-focused assessment.

Aside from the potential for invalid estimates of a child’s cognitive ability, there are other ways failure to first assess for language deficits can lead to diagnostic and treatment errors. For example, a child with an undiagnosed mild-to-moderate receptive language disorder, could be mistakenly misdiagnosed as having ADHD. This misdiagnosis could occur because of language-based difficulty with understanding verbally presented instructions or comprehending class lessons (Hoza et al., 2023), which may be hard to distinguish in a large group classroom setting from several of the DSM-5-TR’s hallmark symptoms of inattention (i.e., “Often has difficulty sustaining attention…,” “Often does not follow through on instructions…”; American Psychiatric Association, 2022, p. 68). Similarly, difficulties with turn taking in social contexts or inappropriately interrupting others could be due to a pragmatic language disorder instead of the impulsivity associated with ADHD. In scenarios such as these, there is the possibility that a child with an undiagnosed language disorder could receive an ADHD diagnosis and undergo a first-line treatment for ADHD (e.g., stimulant medication) without any detection or treatment of the existing or emerging language problems.

Finally, as noted above, social deficits are extremely common in neurodevelopmental disorders. Previous work has demonstrated that language competence is related to social status with peers (Gertner et al., 1994; van der Wilt et al., 2019), although the direction of causality between these variables has not been definitively established (van der Wilt et al., 2019). It is easy to imagine that without a minimal level of language competence, children may be unable to effectively enter peer group interactions, engage in the reciprocal interactions that are critical to friendship formation, or even understand the demands of peer group interactions (Gertner et al., 1994). Without acquisition of these basic language competencies, therefore, social interactions are unlikely to be successful. Accordingly, in our view, language competencies should be screened whenever social difficulties are present.

## 4 The importance of multidisciplinary collaboration

We emphasize here that multidisciplinary collaboration is a necessary component of a transdiagnostic approach (Gillberg, 2010; Neville, 2013; Pham and Riviere, 2015), although it is not often emphasized in routine clinical practice when assessing for neurodevelopmental disorders. Common developmental risk factors (e.g., language delays, behavior problems, motor delays) exist across an array of disorders (e.g., ASD, ADHD, language disorder) that are not all within the wheelhouse of a single discipline. Yet, a comprehensive approach to early intervention would require addressing the full range of difficulties a child is experiencing (Cleaton and Kirby, 2018). Integrating across disciplines that serve children with developmental needs, such as primary care, psychology, psychiatry, speech/language pathology, education, physical therapy, and occupational therapy, undoubtedly will prove challenging, but at the same time, will provide the most comprehensive approach to both understanding suboptimal development and to providing the best care for neurodevelopmental disorders. Therefore, consistent with others’ views on this topic (Gillberg, 2010; Neville, 2013; Pham and Riviere, 2015; Cleaton and Kirby, 2018) we argue here that multidisciplinary collaboration in the assessment and treatment of neurodevelopmental disorders should be a fundamental component of comprehensive care.

Finally, the transdiagnostic dimensional approach allows also for the simultaneous consideration of contextual factors that may influence children’s healthy development. These contextual factors include a wide range of possibilities such as family resources (e.g., economic status, quality of housing, food security, parental educational attainment), environmental resources such as characteristics of the natural and built environments within which families live (e.g., access to green space), access to quality medical care and educational opportunities, and lived experiences such as exposure to racial/ethnic bias or discrimination (National Scientific Council on the Developing Child, 2023). These individual contextual factors likely explain, at least in part, why children exhibiting very similar profiles of developmental impairments may have very different developmental trajectories. As a result, we argue that the scope of multidisciplinary collaborators involved in promoting children’s optimal development extends beyond the health and mental health professions to also include stakeholders from public health and government bodies that legislate and regulate policies regarding access to resources.

## 5 Discussion

Based on the perspectives shared here, we believe there are several needed modifications to our assessment/treatment practices for neurodevelopmental disorders. First and foremost is the need for a multidisciplinary and transdiagnostic framework for identifying delays as early as possible across multiple areas of function (Gillberg, 2010; Neville, 2013; Pham and Riviere, 2015; Cleaton and Kirby, 2018). Given the focus of this special issue on social cognition and discourse processing, we discussed the implications of language competence in social contexts for accurate assessment of neurodevelopmental disorders. However, the need for adequate screening of the other developmental domains is critical as well. The important point is to avoid the temptation to assess primarily for a single disorder in the early stages of an assessment. Instead, it is essential to conduct a comprehensive screening of all basic domains of development to allow for detection of all areas of impairment, not just the one driving the assessment process (Gillberg, 2010; Neville, 2013; Cleaton and Kirby, 2018). This transdiagnostic approach is the only way to fully understand the nature and extent of a child’s problems, allowing for more effective early intervention and treatment.

Second, a multidisciplinary, transdiagnostic framework is ideally suited to a prevention approach as delays can be identified at first signs of dysfunction, well before a child is likely to meet criteria for a disorder (Astle et al., 2022). Early identification of first signs of delay may provide an opportunity to grow the brain during early childhood periods of greatest brain plasticity, through effectively timed early intervention, potentially altering a child’s trajectory toward disorder (Nelson et al., 2023). This strategy is in direct contrast to a “wait-and-see approach”, which we argue, consistent with others (Capone Singleton, 2018), is not appropriate. A proactive early intervention approach is especially critical in domains such as language, since language delays can directly impede development of competence in other areas such as social interaction, emotion regulation, and school readiness. Unfortunately, missed opportunities for early intervention can snowball into multi-domain impairments that persist across the life span (Capone Singleton, 2018).

Third, within child-serving organizations, there are facilitating factors that can be leveraged and barriers that will need to be addressed, to provide the infrastructure and resources necessary to support large-scale screening and early intervention (Peterson-Katz et al., 2023). These factors include the need for organizational support and strong leadership promoting screening practices and allocating sufficient time and organizational resources for practitioners to successfully incorporate screening into daily routines. This might include training opportunities, ongoing support through coaching or group-level discussions on an ongoing basis, and time allocated within the busy workday to allow practitioners to complete the screenings (Meurer et al., 2022; Peterson-Katz et al., 2023). Indeed, it seems unlikely that individual practitioners acting in isolation without a supportive organizational culture will be successful at adopting a transdiagnostic approach to screening. A team approach with shared goals, clear roles and responsibilities, adequate resources, and strong relationships supporting team members seems optimal (Meurer et al., 2022; Peterson-Katz et al., 2023). In addition, Meurer et al. (2022) emphasize the importance of using standardized screening measures and incorporating screening reminders and resulting data and recommendations into electronic medical records, to facilitate easy access to information for all providers serving a child. Yet, despite best intentions, we believe it will be very difficult for practitioners serving children to make the shift toward a transdiagnostic screening approach that prioritizes early detection and early intervention unless there is adequate buy-in from key stakeholders such as insurance companies, school districts, public health officials, and legislators who have the authority to prioritize prevention alongside intervention, and to allocate sufficient resources accordingly. The development of a systems-level approach to addressing developmental delays is our challenge for the future.

Finally, it is important to recognize that contextual factors often contribute to health and mental health inequities both in terms of screening and early intervention. For example, even though improving health equity was explicitly targeted as a goal in their quality improvement project, Meurer et al. (2022) found lower rates of screening for Black children, those on Medicaid insurance, and those living in the lower income neighborhoods. Hence, practitioners and policy makers must prioritize addressing factors beyond the individual child to consider these contextual factors as well as implement strategies to bolster the knowledge and resources of children’s community supports (Meurer et al., 2022). Indeed, addressing systemic factors that serve as barriers to the ongoing monitoring of all young children’s development is critical for promoting health and mental health equity.

## Data availability statement

The original contributions presented in the article are included in the article/supplementary material, further inquiries can be directed to the corresponding author.

## Author contributions

BH: Writing – original draft, Writing – review & editing, Conceptualization. ES: Writing – review & editing, Conceptualization.
